# ReprOlive: a database with linked data for the olive tree (*Olea europaea* L.) reproductive transcriptome

**DOI:** 10.3389/fpls.2015.00625

**Published:** 2015-08-11

**Authors:** Rosario Carmona, Adoración Zafra, Pedro Seoane, Antonio J. Castro, Darío Guerrero-Fernández, Trinidad Castillo-Castillo, Ana Medina-García, Francisco M. Cánovas, José F. Aldana-Montes, Ismael Navas-Delgado, Juan de Dios Alché, M. Gonzalo Claros

**Affiliations:** ^1^Department of Biochemistry, Cell and Molecular Biology of Plants, Estación Experimental del Zaidín, Consejo Superior de Investigaciones CientíficasGranada, Spain; ^2^Plataforma Andaluza de Bioinformática, Edificio de Bioinnovación, Universidad de MálagaMálaga, Spain; ^3^Departamento de Biología Molecular y Bioquímica, Facultad de Ciencias, Universidad de MálagaMálaga, Spain; ^4^Departamento de Lenguajes y Ciencias de la Computación, Universidad de MálagaMálaga, Spain

**Keywords:** olive, transcriptome, reproduction, database, pollen, pistil, annotation

## Abstract

Plant reproductive transcriptomes have been analyzed in different species due to the agronomical and biotechnological importance of plant reproduction. Here we presented an olive tree reproductive transcriptome database with samples from pollen and pistil at different developmental stages, and leaf and root as control vegetative tissues http://reprolive.eez.csic.es). It was developed from 2,077,309 raw reads to 1,549 Sanger sequences. Using a pre-defined workflow based on open-source tools, sequences were pre-processed, assembled, mapped, and annotated with expression data, descriptions, GO terms, InterPro signatures, EC numbers, KEGG pathways, ORFs, and SSRs. Tentative transcripts (TTs) were also annotated with the corresponding orthologs in *Arabidopsis thaliana* from TAIR and RefSeq databases to enable Linked Data integration. It results in a reproductive transcriptome comprising 72,846 contigs with average length of 686 bp, of which 63,965 (87.8%) included at least one functional annotation, and 55,356 (75.9%) had an ortholog. A minimum of 23,568 different TTs was identified and 5,835 of them contain a complete ORF. The representative reproductive transcriptome can be reduced to 28,972 TTs for further gene expression studies. Partial transcriptomes from pollen, pistil, and vegetative tissues as control were also constructed. ReprOlive provides free access and download capability to these results. Retrieval mechanisms for sequences and transcript annotations are provided. Graphical localization of annotated enzymes into KEGG pathways is also possible. Finally, ReprOlive has included a semantic conceptualisation by means of a Resource Description Framework (RDF) allowing a Linked Data search for extracting the most updated information related to enzymes, interactions, allergens, structures, and reactive oxygen species.

## Introduction

Research in plant reproduction is accelerating rapidly as a direct consequence of the technological progresses (Dickinson and Franklin-Tong, 2011). Differential screening was initially used to identify abundant or specific transcripts of very specialized cells (Engel et al., 2003) and have been progressively replaced by the use of commercial microarrays and RNA-sequencing platforms. The use of transcriptomic approaches is unraveling the particular functionality of the key subsets of cells in charge of male and female gamete formation, and the complex interactions and signaling networks involved in the pollen–pistil interaction [reviewed in (Dukowic-Schulze and Chen, 2014)]. In good agreement with the degree of difficulty in isolating reproductive cells and extracting their RNAs, most studies up to date have reported gene expression in whole anther tissues, followed by male meiocytes, and female structures and meiocytes, being *Arabidopsis thaliana* the most widely studied, followed by lily, tobacco, brassica, petunia, maize, cotton, rice, and others (Dukowic-Schulze and Chen, 2014; Rutley and Twell, 2015). Those studies had shown that reproductive transcriptomes are substantially different from their vegetative counterparts, in concordance with the high proportion of specific transcripts present in these tissues, sometimes 4–11% of the total number of the genes expressed, depending on the normalization algorithms used and the number and diversity of sporophytic data sets used for comparison (Rutley and Twell, 2015). Moreover, huge differences in terms of temporal expression are present among developmental stages (e.g., meiosis initiation, mature pollen, pollen germination) and spatial/tissue localization (e.g., sporogenous tissue, tapetum, isolated pollen grains, pollen tube…), the most striking changes occurring in the mature pollen upon hydration and germination (Wang et al., 2008; Wei et al., 2010). The peculiarity of reproductive tissues in terms of gene expression also deserves a dedicated study not only for agronomical, biological, and biotechnological reasons but also in search of putative new allergens in pollen (El Kelish et al., 2014; Villalba et al., 2014).

Olive tree (*Olea europaea* L.) is one of the most important oil-producing plant species all over the world. While waiting for the genome sequence (Muleo et al., 2012), transcriptomic approaches have been exploited. For example, subtractive libraries from olive fruits sampled at three different stages shed light on metabolic pathways and transcriptional aspects related to carbohydrates, fatty acids, secondary metabolites, transcription factors, and hormones as well as response to biotic and abiotic stresses throughout olive drupe development (Galla et al., 2009). Comparative 454 pyrosequencing from two olive genotypes during fruit development provided information about the structure and putative function of gene transcripts accumulated during fruit development, reporting differentially expressed genes with potential relevance in regulating the fruit metabolism and phenolic content during ripening (Alagna et al., 2009). ESTs were generated from two cDNA libraries from young olive leaves and immature olive fruits (Ozgenturk et al., 2010), which serve as a valuable source for further functional studies. Sanger sequencing and further microarray analysis identified differentially expressed transcripts in salt–tolerant and salt–sensitive olive cultivars (Bazakos et al., 2012). The olive abscission zone during cell separation in order to understand mature fruit abscission control was also studied by high-throughput sequencing (Gil-Amado and Gomez-Jimenez, 2013) to help in current olive breeding programs. More recently, 12 cDNA libraries from olive fruit, seeds, young stems, leaves, buds, and roots were sequenced, assembled and annotated (Muñoz-Merida et al., 2013). It is quite promising that information about olive genome is appearing in recent years. For example, The Olive Genome Project (OLEA) is expected to offer transcriptomic studies, molecular markers and genomic information about the Leccino cultivar^1^. There is also the International Olive (*O. europaea*) Genome Consortium (IOGC) whose goal is to sequence the whole genome of olive and identify the biological nature of this plant^2^. The current status of a wild olive sequencing an annotation can be downloaded from IOGC, and several basic, genome analyses have been implemented on the web page.

A number of questions involving olive reproductive biology are still open. They include the search of explanations and the definition of criteria for potential improvements of the plant as regard to the selection of genotypes, the culture conditions to prevent alternate bearing [the tendency for not to bear a regular and similar crop yield year after year (Turktas et al., 2013)], the extended juvenility of the plant (particularly in some cultivars), and the presence of self-incompatible genotypes. Knowledge about the pollen-pistil interactions in this plant is still scarce, and molecular evidence of the presence of self-incompatibility mechanisms (although largely suspected of the gametophytic type), is also limited in spite of the most recent transcriptomic analyses reported as conference proceedings (Barcaccia et al., 2012; Collani et al., 2012). Hence, this study extracted RNAs from pollen and pistil in different maturing and developing stages to provide a reproductive transcriptome of olive tree and a user-friendly database containing the resulting information. Database queries may help scientists to develop further research and to design strategies to improve both yield and quality in these agronomic fields. Moreover, new clinical approaches are also expected to derive from the increased knowledge about the putative allergens present in the olive pollen.

## Materials and Methods

### Sequence Processing

#### RNA Sources and Sequencing

With the aim of providing sequences from the development of olive reproductive tissues, eight gene libraries were constructed (**Table 1**). RNAs and mRNAs from mature pollen grains, *in vitro* germinated pollen at two different times after hydration (1 and 5 h), and pistils at developmental stages 2, 3, and 4 [as defined by (Zafra et al., 2010)] were isolated using RNeasy Plant and Oligotex PolyA+ kits (Qiagen), respectively. cDNA libraries to be sequenced with a Roche GS-FLX Titanium+ were generated using the cDNA Synthesis System Kit (Roche) and the raw read were uploaded to the SRA database with BioProject ID PRJNA287107^3^. As a representation of olive vegetative transcriptome for control purposes, four additional gene libraries from olive leaves, roots, and radicles were constructed and sequenced. Finally, the three subtractive libraries (named with “Subs” in **Table 1**) resulting from the comparison of mature pollen, pistils at developmental stage 4 and leaves, sequenced by the classical Sanger method (Zafra et al., in preparation), were also included.

**Table 1:** Gene libraries used in ReprOlive.

Gene library	Tissue	Developmental stage	Sequencing method	Raw reads	Useful reads
PM-Subs	Pollen	Mature	Sanger	666	518
PM	Pollen	Mature	Pyrosequencing	216,497	111,242
PG1	Pollen	1 h germination	Pyrosequencing	258,167	141,232
PG5	Pollen	5 h germination	Pyrosequencing	233,921	120,276
S2	Pistil	Stage2	Pyrosequencing	257,813	138,077
S3	Pistil	Stage3	Pyrosequencing	247,401	141,903
S4	Pistil	Stage4	Pyrosequencing	262,269	149,929
S4-Subs	Pistil	Stage4	Sanger	480	256
L	Leaf	Mature	Pyrosequencing	223,399	41,178
L-Subs	Leaf	Mature	Sanger	403	251
R1	Root	Mature	Pyrosequencing	231,237	25,899
R2	Root	Radicle	Pyrosequencing	145,204	22,075

#### Sequence Pre-Processing and Assembling

Raw reads were pre-processed and assembled following the same pipeline as previously described by our laboratory (Benzekri et al., 2014; Canales et al., 2014) and illustrated as a flow diagram in **Supplementary Figure S1**. Briefly, pre-processing was based on SeqTrimNext^4^ (Falgueras et al., 2010) to remove low quality, ambiguous and low complexity stretches, linkers, adaptors, vector fragments, organelle DNA, polyA/polyT tails, and contaminated sequences while keeping the longest informative part of the read. Pyrosequences below 40 bp and Sanger sequences below 100 bp were also discarded. Useful reads (**Tables 1** and **2**) were assembled with an overlap-layout-consensus algorithm such as MIRA3 (Chevreux et al., 2004), and a strict de Bruijn graph analyzed by a Eulerian path such as Euler-SR (Pevzner et al., 2001). The contigs obtained (**Table 2**) were reconciled with CAP3 (Huang and Madan, 1999) at 85% similarity to provide a final set or tentative transcripts (TTs) having consensus sequences closer to real transcripts (Liang et al., 2000; Fernandez-Pozo et al., 2011). The overestimated number of TTs in these tissues was reduced on the basis of TT annotations.

**Table 2:** Main features of transcriptomes in ReprOlive based on Full-LengtherNext analyses.

Feature	Pollen	Pistil	Vegetative	Reproductive
**Assembling statistics**				
Number of useful reads	373,268	430,165	89,403	803,433
Mean length (nt)	383	385	545	384
Number of MIRA3 contigs	54,754	73,823	42,310	116,298
Number of Euler-SR contigs	4,807	15,216	490	16,211
Number of Contigs after CAP3 reconciliation	28,094	60,964	39,425	73,589
Number of TTs without chimeras and artifacts^∗^	27,823	60,400	38,919	72,846
Mean length (nt)	608	678	664	686
N50 (nt)	661	780	683	798
**Annotation statistics**				
Longest TT (nt)	7,016	7,757	2,865	7,950
Number of ncRNAs	31	17	265	45
Number of TTs with annotation	24,861	54,129	36,700	63,965
Number of TTs with ortholog	21,607	46,910	32,076	55,356
With unique ortholog IDs	11,672	21,326	15,003	23,568
With ortholog from *Arabidopsis thaliana* RefSeq	21,233	46,924	31,945	54,890
Unique RefSeq IDs	9,769	16,565	12,489	17,612
With ortholog from *A. thaliana* TAIR10	21,312	47,038	31,980	55,067
Unique TAIR10 IDs	8,922	14,656	11,247	15,503
Number of TTs coding a complete protein	2,809	7,137	3,559	9,157
Unique, complete proteins	1,976	4,822	2,220	5,835
Number of TTs without ortholog	6,185	13,473	6,578	17,445
Likely coding for a complete protein	170	446	242	628
Likely coding for an incomplete protein	2610	5,312	2,523	6,486
**Reference transcriptome**				
Number of representative TTs	13,589	25,720	17,340	28,972
*Arabidopsis thaliana* RefSeq orthologs	10,878	20,612	14,576	22,565
Unique RefSeq IDs	8,281	13,901	10,349	14,706
*Arabidopsis thaliana* TAIR10 orthologs	10,900	20,658	14,581	22,638
Unique TAIR10 IDs	7,842	12,883	9,756	13,584

*^∗^Artifacts are internal, direct or inverse, repetitions.*

#### Annotation

Functional classification of a list of interesting genes is absolutely required for future comparative studies. Reliable annotations were generated by combining separate information sources. Therefore, gene descriptions (taken from the closest plant ortholog), GO terms, Enzyme Commission codes (ECs), and InterPro signatures were provided by Sma3s (Muñoz-Mérida et al., 2014) using the non-redundant plant division of UniProtKB in order to remove spurious annotations. KEGG maps were retrieved directly from the KEGG site using the obtained ECs. Another gene description (based on the closest plant ortholog >45% identical), putative start and stop codons, predicted amino-acid sequence, ORF status (full-length or incomplete coded proteins), putative ncRNAs (based on fRNAdb sequences^5^) excluding mature miRNA and other short reads], *A. thaliana* ortholog from TAIR10 (Lamesch et al., 2012) and RefSeq (Pruitt et al., 2012; as is in Nov 2012), protein coding status based on TransDecoder^6^ (Haas et al., 2013), and the reference set of TTs were provided by Full-LengtherNext^7^ (Seoane et al., submitted). Microsatellites, as a source of genetic markers, were obtained by screening for the presence of SSR motifs using MREPS^8^ (Kolpakov et al., 2003) with default parameters counting repeats whose period was at least 2 and size at least 12 and a coverage of up to 1000 reads. A total of 5,835 reproductive TT (1,976 in pollen and 4,822 in pistil transcriptomes, **Table 2**) are having microsatellites on their sequences. In our previous experience (Fernandez-Pozo et al., 2011; Benzekri et al., 2014; Canales et al., 2014), detection of SNPs in natural populations requires a huge amount of data and is very difficult to interpret (Benzekri et al., 2014); therefore, SNPs have not been predicted.

The flow template based on AutoFlow (Seoane et al., in preparation) that automates the complete process from pre-processing to annotation is detailed in **Supplementary Figure S2**.

#### Expression Data

Since the Roche FLX platform produces a limited number of reads in contrast to Illumina ultrasequences, libraries described in **Table 1** were combined to obtain a pool of reads from pollen (libraries PM-Subs, PM, PG1 and PG2, 373,268 reads), pistil (libraries S2, S3, S4, S4-Subs, 430,165 reads) and vegetative tissues (libraries L, L-Subs, R1 and R2, 89,403 reads). These reads were mapped to all reference TT included in ReproOlive (**Table 2**) using Bowtie2 (Langmead and Salzberg, 2012) and allowing each read to map in every complementary TT. Mapped reads were counted with Bio-samtools from BioRuby (Goto et al., 2010) and included in the database as row counts (available for download to be analyzed with other software) or as RPKM values (for comparing purposes in order to clearly identify TTs specific or not from pollen and/or pistil).

### Database Construction

#### Implementation and Architecture

**FIGURE 1:**
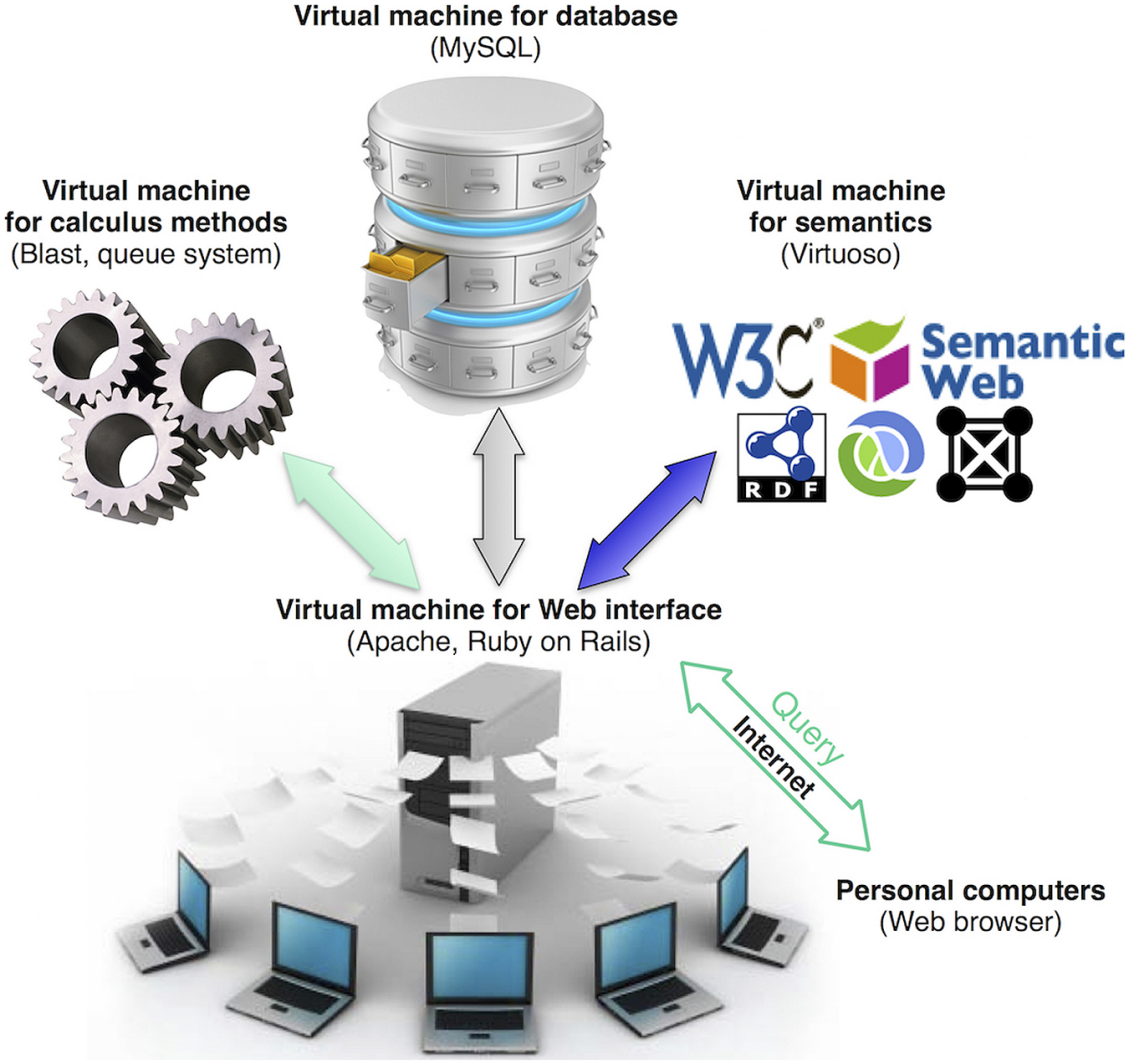
**Database organization in virtual machines.** Using Web browsers, users will retrieve data from ReprOlive by querying or browsing through the Web interface. Web pages are generated on demand by the virtual machine that manages the Web interface. When queries require intensive calculations (for example, Blast comparisons), they are performed by the virtual machine for calculus. Queries to the RDF of ReprOlive are handled by the virtual machine for semantics. The use of different virtual machines is transparent for the user that can only see inputs and outputs in user’s Web browser.

ReprOlive runs with the Apache HTTP Server 2 and MySQL 5 database management system in Linux OS. Ruby On Rails^9^ 2.3.11 scripts were used to create the user interface on HTML 5 coupled with MySQL to use of a model-view-controller pattern to maintain strict separation between the web interface (views) code, database contents (models), and all methods that handle interactions between views and database (controllers). This allowed to divide the database in four different virtual machines (**Figure 1**): one for the web interface, one for the database content, one for calculus methods (e.g., blast queries) and the fourth for Linked Data (semantic) search. BioRuby (Goto et al., 2010) is required for some importation and managing tasks. The functionality of the Linked Data search was implemented using a SPARQL EndPoint [a service to send queries to the Resource Description Framework (RDF) database] provided by an instance of Virtuoso Open-Source Edition. RDF information has been produced using D2RQ (dump-rdf script), mapping the database schema with one application ontology^10^. The use of independent virtual machines distributes tasks between machines, allowing for multiple, concomitant browsing and searching capabilities.

#### Availability and Updates

ReprOlive is freely available at http://reprolive.eez.csic.es. Bulk imports, updates, and database managements were automated: when source data are saved in *import_new_projects* folder, the database automatically launches the necessary Ruby gems that import sequences, annotations, and expression data into a new assembly version of the database. Therefore, updates of ReprOlive transcriptomes with re-assembled and re-annotated TTs, and new expression data, will be automatically incorporated, making the database easily scalable, maintainable, and expandable. Implementation based on independent virtual machines makes ReprOlive easily clonable and adaptable to any computer environment without complicated installations.

## Results and Discussion

### Web Interface and Navigation

**FIGURE 2:**
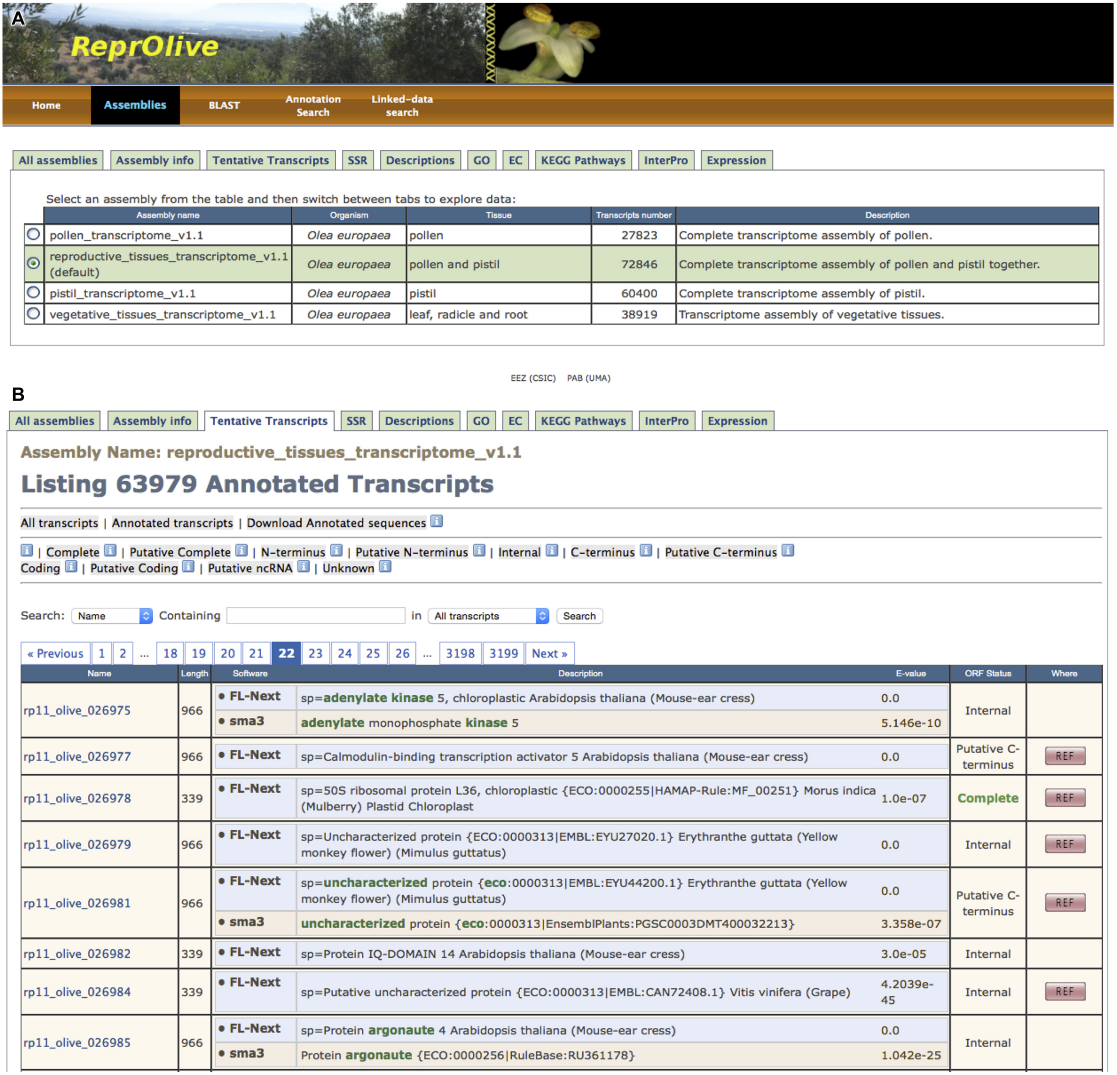
**Screen captures providing a general overview of ReprOlive. (A)** “All assemblies” panel showing the four transcriptomes included in the database. **(B)** First page of “Tentative transcript (TTs)” panel, where filtering criteria are accessible and information about each TT is displayed (see text for details). TTs are shown in pages of 20 sequences.

Since molecular sequence databases are fundamental resources for modern bioscientists, ReprOlive currently houses annotated sequences of olive tree pollen, pistil, a small set of vegetative tissues, and a tentative transcriptome combining reproductive tissues (**Table 2** and **Figure 2A**). It has been designed with a user-friendly interface that can be browsed anonymously to facilitate researchers to access to this information. There is a navigation bar containing buttons for database mining from different entry points and based on different criteria, including three different possibilities of search (by sequence, by text strings on annotations, and by Linked Data).

#### Home Page

ReprOlive is accessed by a home page where general information is offered in three panels. The left panel contains links to the version history of the database, the scheme of the pipeline that produced the last version as automatically provided with AutoFlow, and the history of visits. On the right panel, information about tool versions used in the assembly pipeline, the current database release and the funding credits. This page can be recalled by means of the “Home” button in the navigation bar.

#### Assemblies

The button “Assemblies” in the navigation bar (**Figure 2A**) opens the page to start browsing the database by means of tab panels (**Figure 2A**, from “All assemblies” to “Expressions”, all explained in next subsections); note that the word ‘assemblies’ is used here as synonym of ‘transcriptomes.’ The “All assemblies” tab is shown by default when “Assemblies” is selected in the navigation bar. This tab displays the four transcriptomes that can be browsed in ReprOlive: pollen, pistil, reproductive (includes the sum of pistil and pollen data but not both tracks separately) and vegetative (includes the sum of root and leaf data but not both tracks separately; **Figure 2A**). The reproductive transcriptome is selected by default (**Figure 2A**) but any of the other three can be selected by the scientist. Multiple selections of transcriptomes are not allowed since only one transcriptome can be browsed at a time.

The tab “Assembly info” will provide general and statistical information about the transcriptome selected in the “All assemblies” tab. The name of the selected transcriptome is always shown in the first row of any as Assembly Name (**Figure 2B**). The page is vertically divided, where the left side contains detailed information about the chosen assembly. The right side gives the possibility of (i) downloading the whole set of TTs in Fasta format, (ii) downloading only the reference TT with annotations, (iii) downloading sequence and annotations for a custom set of TT identifiers, and (iv) downloading raw expression data. These capabilities have been designed to provide data for further use in external tools instead of embedding cumbersome, bioinformatic tools in the own database.

#### Tentative Transcripts’ Tab

To navigate through all TTs in the assembly (transcriptome) in a paginated way, the tab of “Transcripts” in the navigation bar must be selected (**Figure 2B**). Each TT code is illustrated with relevant information, such as its length, descriptions, ORF status, and if it is a reference TT (column ‘Where’). Since consistency of descriptions is a sign of reliable annotation, common words in the description for one TT are marked in green. There are three independent ways of filtering TTs (identifiable in different rows in **Figure 2B**) that are always applicable on the complete list of TTs:

(1) The first filtering of TTs can be done with the first row of three gray buttons (**Figure 2B**). By default, the view of “Annotated transcripts” (second button in the row) is displayed, but all TTs can be shown using the “All transcripts” (first button in the row).
(2) The second filtering corresponds to the second row of gray buttons (**Figure 2B**, from “Complete” to “Unknown”) that correspond to ORF statuses. For example, “Complete” gray button filters out TTs that do not code for a complete protein; “Coding” button retains TTs without predicted ORF that should code for a protein based on the TransDecoder test. Also, TTs corresponding to putative ncRNA precursors can be selected with the corresponding “Putative ncRNA” gray button of this row.
(3) The third way of filtering is based on the text content of TTs by means of pop-up menu and text field below the previous rows (**Figure 2B**). This filtering allows finding a particular TT (e.g., the cysteine proteinase coded by ‘rp11_olive_018645’), or a family of sequences (e.g., for the reproductive transcriptome, the 115 cysteine proteinases in ReprOlive, or the 16 complete ORFs coding for cysteine proteinases).

**FIGURE 3:**
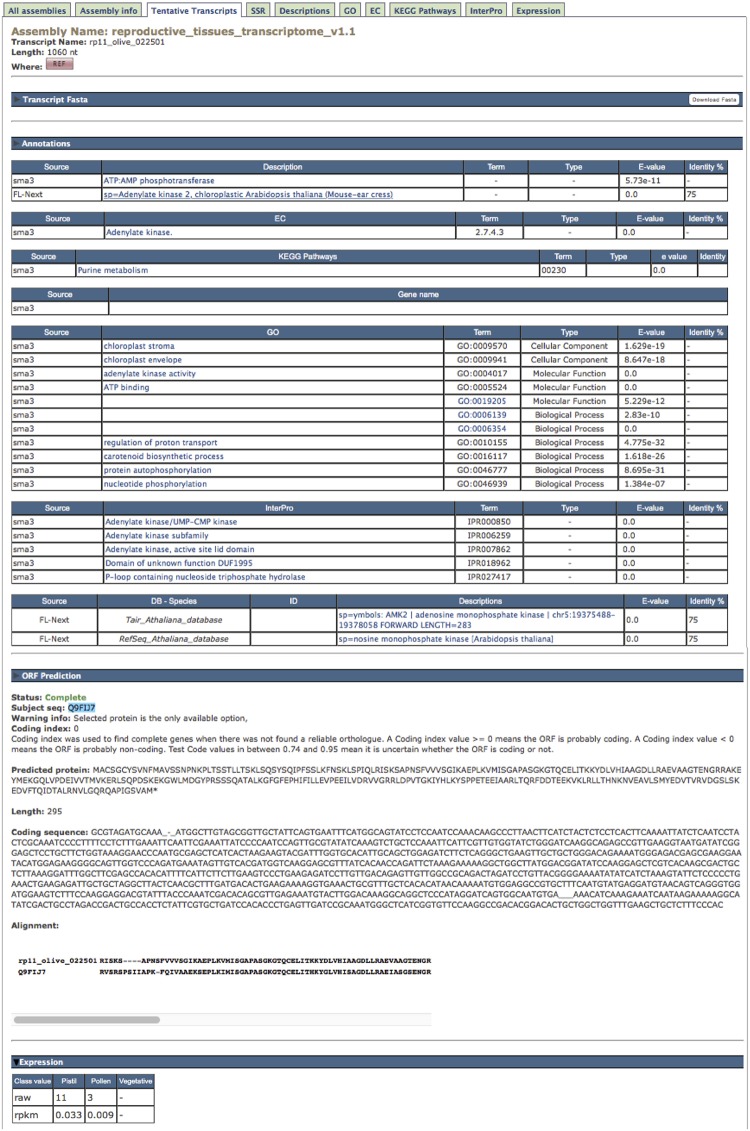
**Information shown for one single TT, i.e., ‘rp11_olive_022501’ corresponding to an adenylate kinase where the “Annotation” and “ORF prediction” pop-up texts are deployed.** See text for details.

Clicking on one TT identifier (column “Name”, **Figure 2B**) in any displayed list, the complete information about this TT is shown as a pop-up text (**Figure 3**). The first block, named “Transcript Fasta,” shows the sequence and a button on the right to download it in Fasta format. The “Annotations” block contains the following tables: (1) the assigned descriptions by Sma3s and Full-LengtherNext as a user-friendly way to offer information about tentative functions; (2) tables for gene names, GOs, ECs, KEGG pathways, each one accompanied by the associated *E*-value to enable another empirical assessment of annotation quality. (3) the InterPro signatures, which add high-valued annotations, such as functional sites, protein families, or conserved domains, with a single search (Hunter et al., 2012); and (4) the TAIR and RefSeq orthologs of *A. thaliana* (**Table 2**), permitting gene-enrichment and functional analyses with a non-model species such as olive tree. The “ORF prediction” block (**Figure 3**) comes from the Full-LengtherNext predictions, providing the putative ORF, if this ORF is complete, the position of start and/or stop codons, and the alignment that allows such predictions. The ORF prediction is an extremely useful information that will find direct use in laboratories, for example in designing primers to clone ORFs, or designing 3′-probes that discern between closely related TTs. Finally, if the TT shown is part of the reference transcriptome, the “Expression” block at the bottom will show the raw counts and RPKM of this TT in the three types of tissues included in ReprOlive (pollen, pistil, and vegetative). These data display whether the expression of this TT is specific, up-regulated or down-regulated in any of the samples. For example, the TT shown in **Figure 3** is more expressed in pistil than in pollen, and is not expressed in vegetative (leaf and root) tissues, making it a good candidate for a specific reproductive TT.

#### SSR Tab

Plant cDNAs from natural orchards should be heterozygous and contain a high frequency of polymorphisms. Since microsatellites (SSRs) occur frequently in most eukaryote genomes and can be very informative, multi-allelic and reproducible, the “SSR” tab (**Figure 2**) shows the list of TTs having at least one SSR available and the TT where it is found. Each SSR motif is shown as a tetrad containing its sequence, the TT that contains it, and the start and end positions. SSRs can be filtered by the number of nucleotides in the motif and by their length, revealing that reproductive transcriptome has hexanucleotide motifs in 501 TTs, tetranucleotide motifs in 575 TTs, and that 493 SSRs have more than 20 nt in length. SSRs have direct applications as molecular markers since they are easily converted in primers (Guerrero et al., 2010) that provide co-dominant and stable results (Abdellatif and Khidr, 2010) that overcome the limitations of other types of molecular markers (Garcia et al., 2004). Moreover, ORF-based SSRs are more advantageous since they will reduce the mapping efforts required for the development of high-density maps and association studies, and will facilitate comparative genomics.

#### Descriptions, GO, EC, and InterPro Tabs

**FIGURE 4:**
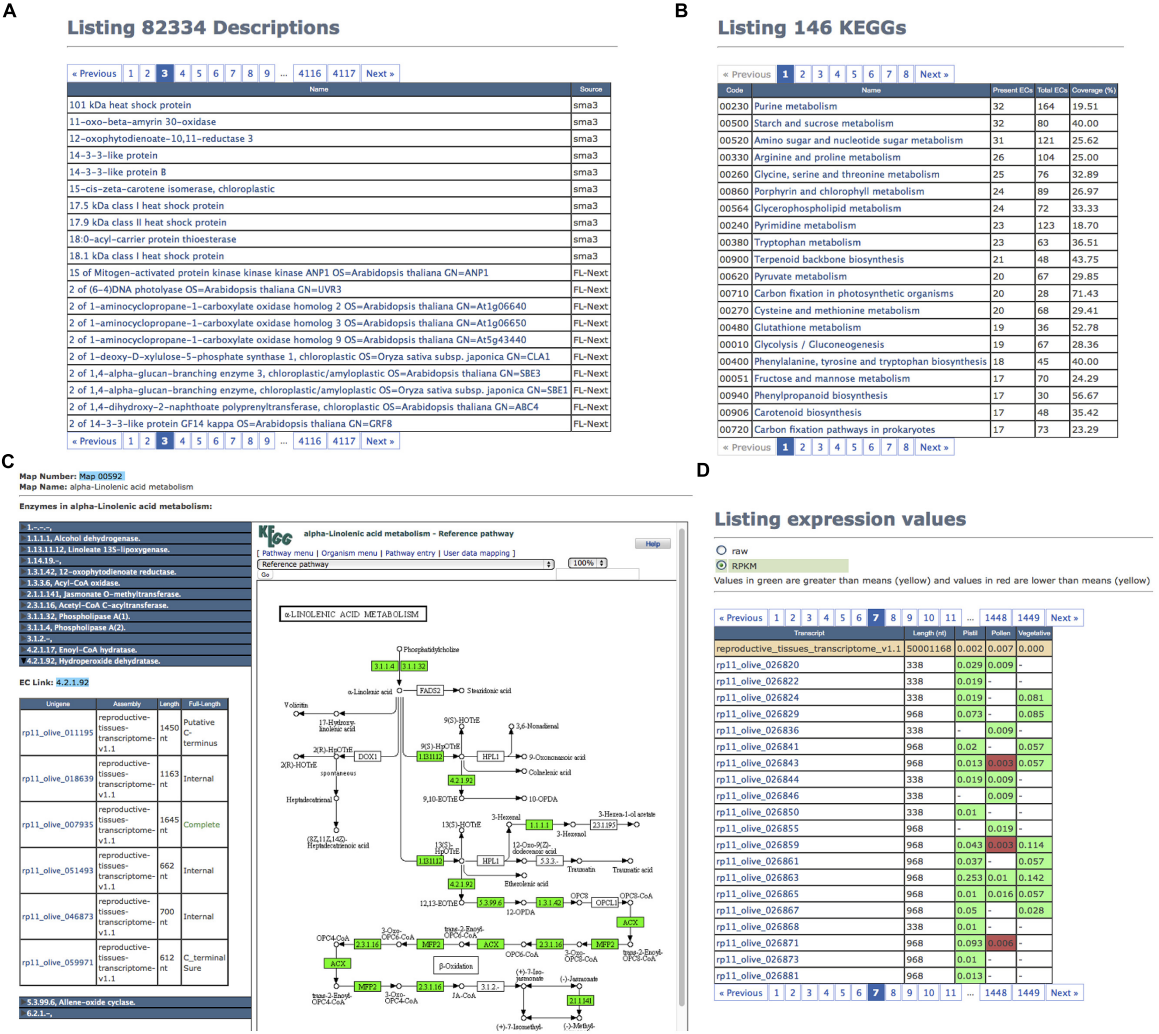
**Reproductive transcriptome illustrating the views of Description, KEGG Pathways, and Expression tab panels. (A)** List of TT description that illustrates the lack of consistence between descriptions provided by humans. **(B)** First page of KEGG Pathways showing the most populated ones in ReprOlive. **(C)** Example of α-linolenic acid metabolism pathway where enzymes in ReprOlive are highlighted in green; the TTs corresponding to 4.2.1.92 are deployed to show that there is at least one coding for the complete protein. **(D)** The seventh page illustrating different types of TT expression based on RPKMs.

The TT annotations (**Figure 3**) can also be browsed by means of their respective tab panels (**Figure 2**). Unfortunately, since descriptions were written by humans, it is frequent to find different descriptions for the same sequence (**Figure 4A**), as can be deduced by the fact that there are 82,334 descriptions for 63,965 TTs with functional annotations. Clicking on one description, the collection of TTs sharing it is displayed. Tab panels (**Figure 2**) for browsing through the 45,781 GOs, the 10,003 ECs, and the 187,899 InterPros behave as in the “Description” tab panel (**Figure 4**).

#### KEGG Pathways’ Tab

**FIGURE 5:**
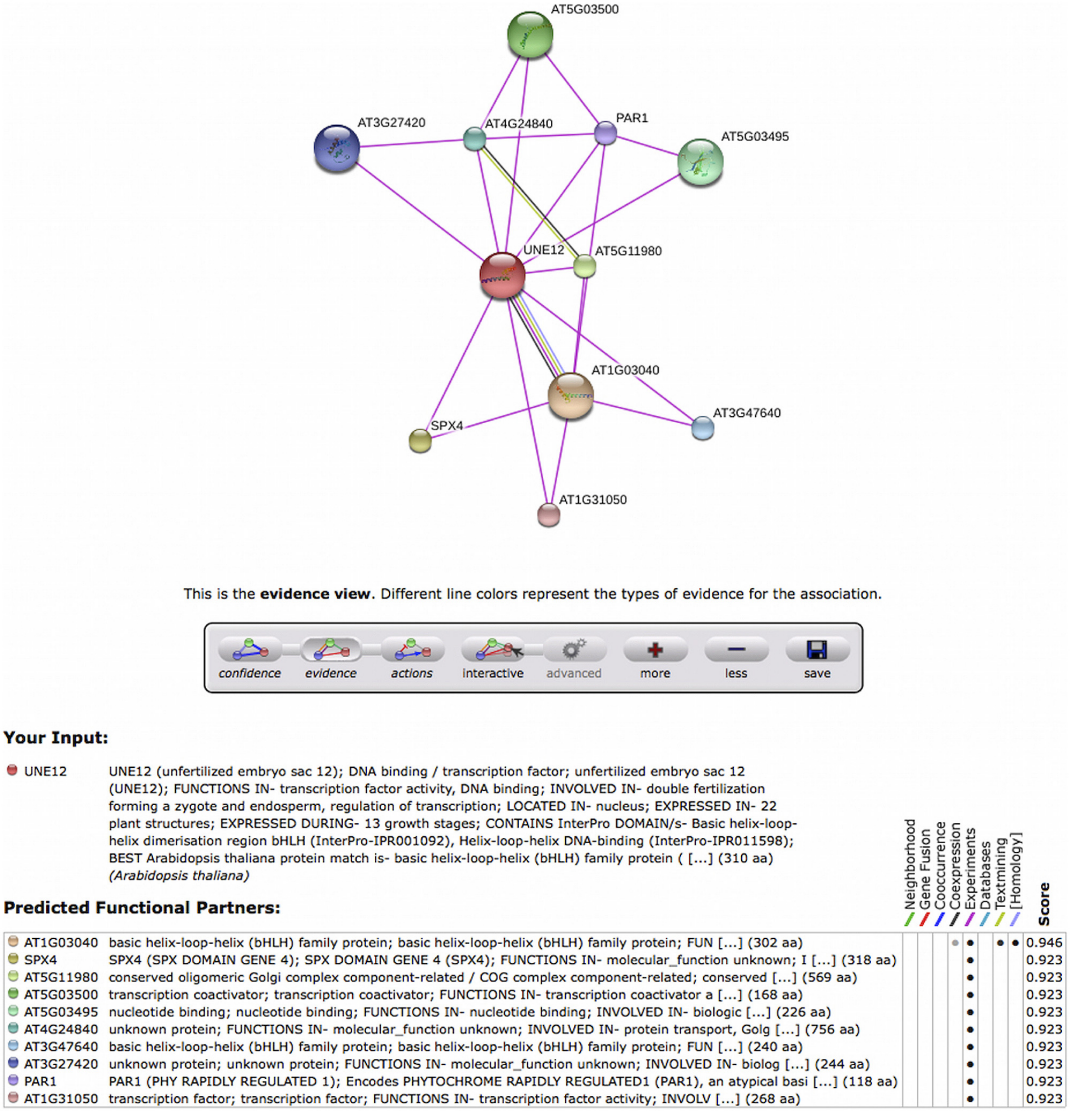
**Screen capture of the evidence view of interactions of UNE12 *Arabidopsis* ortholog to rp11_olive_029403 TT.** This information can be shown in page http://string-db.org/newstring_cgi/show_network_section.pl?identifier=3702.AT4G02590.1-P at the STRING 9.1 database. The magenta color of edges indicate that interactions were reported experimentally. The meaning of each node is explained at the figure bottom.

The “KEGG Pathways” tab panel (**Figures 2** and **5**) shows in a paginated way the 146 reproductive tissue pathways (143 in pollen and 146 in pistil) and 145 vegetative pathways sorted by the number of ECs (**Figure 4B**, “Present ECs” column) identified in ReprOlive. The pathway codes, the total number of pathway enzymes (**Figure 4B**, “Total ECs” column) as well pathway coverages [**Figure 4B**, “Coverage (%)” column], are also displayed. Among the most covered pathways in reproductive transcriptome are 91% coverage of glucosinolate biosynthesis (map 00966), 80% coverage of betalain biosynthesis (map 00965), 75% coverage of brassinosteroid biosynthesis (map 00905), and DDT degradation (map 00351), 71% coverage of carbon fixation (map00710), 68% coverage of α-linolenic acid metabolism (map 00592), and 56% coverage of flavonoid biosynthesis (map 00941) and phenylpropanoid biosynthesis (map 00940).

When clicking on a pathway name, the panel contents change to show the EC list present in ReprOlive for that pathway, and its image displaying in green the enzymes found in the database (**Figure 4C**). The EC names on the left can be deployed to show which ReprOlive TTs have this function assigned. This allows the selection for specific TTs coding the complete protein, such as ‘rp11_olive_007935’ for 4.2.1.92 (hydroperoxide dehydratase). The colored pathway image is interactive and allows the navigation to the KEGG database to obtain more information.

#### Expression Tab

The list of reference TTs with the raw number of reads mapped in this TT or the RPKM as a comparable value can be seen in the “Expression” tab panel (**Figures 2** and **4D**). The first line makes reference to the total number of reads used to map and the total length of the transcriptome to obtain a mean RPKM (total number of reads by the total length of the reference transcriptome) to color RPKM values in green to indicate that it is over the transcriptome mean, and in red when it is below this mean. These values must only be considered illustrative, since RPKM is not the best normalization measure (Wagner et al., 2012), and since RNA-seq values correspond to pyrosequence mapping (and not short read mapping). However, this value can help in determining the tissue where the TT is preferentially expressed. For example, the rp11_olive_005693 (a POZ/BTB domain containing protein) is expressed in the three tissues, mainly in pistil; the pollen seems to specifically express a significant proportion of uncharacterised proteins, such as rp11_olive_028897, and a probable hydrolase (rp11_olive_026947) seems to be expressed only in reproductive tissues, mainly in pollen. Raw expression data are downloadable from the “Assembly Info” tab panel (**Figure 2**).

The utility of ReprOlive annotations together with the expression values expressed as RPKM values is shown in **Supplementary Figure S3**, where the 1,655 TTs that are expressed only in pollen (as per their RPKM) and have a valid RefSeq ortholog have been analyzed using GOrilla (Eden et al., 2009) using the default parameters. The enriched GO terms of the pollen-specific TTs correspond to pollen tube growth, actin filament organization, plant cell wall organization and modification, polysaccharide catabolic process, and carbohydrate transport, to cite the most representative biological processes. Considering the cell component, they are mainly expressed in the extracellular region, followed by the pollen tube and the plant cell wall. These results support the experimental procedure of the sequenced libraries, the assembly, the ortholog annotation and even the RPKM values in spite of they are calculated from Roche/454 reads.

#### Blast Search

Tentative transcripts can be retrieved by sequence similarity using Blast+ (Camacho et al., 2009). A blast-based search engine with customisable *E-*value for nucleotide (blastn) or amino acids (blastx) has been implemented and can be accessed by the button “BLAST” in the navigation bar (**Figure 2**). The type of sequence (amino acid or nucleotides) is automatically detected. Blast searches are conducted against the transcriptomes selected by the corresponding checkbox. Blast executions are queued and the URL where the final result will be stored for a month is shown when the task is finished. The user can download the results as an HTML file (the same information shown on screen) or as the direct blast output.

#### Annotation Search

ReprOlive includes a text-based search that can be accessed by the button “Annotation Search” in the navigation bar (**Figure 2**). It complementary to the filtrations by words in descriptions, by the size of SSR, by the ORF status, etc., since it searches the database by combining GOs, ECs, InterPros, descriptions, orthologs, and gene names. The text strings can be combined using AND or OR. The text-based search can be restricted to only one of the transcriptomes in ReprOlive by means of checkboxes. As a result, a paginated list of TT fulfilling the requirements is shown and TT sequences can be downloaded in Fasta format.

#### Linked Data Search

A novelty of ReprOlive with respect to other plant databases is that its information has also been published as structured in RDF format. This allows its interlinking with other semantic databases, such as UniProtKB. Since most TTs in ReprOlive (mainly the reference transcriptome) have also been annotated with an *Arabidopsis* ortholog on TAIR10 or RefSeq, the Linked Data search can retrieve information about 3D-structures (PDB database), allergens (Allergome database), interactions (STRING and IntAct databases) and enzyme data (BRENDA, BioCyc, KEGG, and Reactome databases). The advantages of this semantic search are gaining access to updated information of external databases, and complementing and extending the stored information in ReprOlive.

Semantic capability of ReprOlive can be accessed by the button “Linked Data Search” in the navigation bar (**Figure 2**). It starts with the automatic selection of which TTs will be the semantic seed. The first row enables to collect TTs sharing IDs, GOs, ECs, or InterPro codes. Clicking on the button “Get local data,” related information on ReprOlive is provided. But clicking on “Search,” the *Arabidopsis* ortholog of every TT is extracted and used to recover external information concerning structures, interactions, allergens, or enzyme data. Retrieved information can be saved using the “Download results” button.

An example of use can start from the rp11_olive_029403 TT that is only expressed in both reproductive tissues and not in vegetative tissues. It is annotated as a transcription factor similar to UNE12 in *Arabidopsis* and with the GO:0080147 corresponding to root hair cell development. Using this GO as seed, seven *Arabidopsis* protein networks (only two in pollen transcriptome) were recovered from STRING v 9.1 database, one of the interaction networks centered on the UNE12 gene of *Arabidopsis* (**Figure 5**) UNE12 is known to be a protein of unfertilized embryo sac involved in double fertilization forming a zygote and endosperm. In addition, this *Arabidopsis* ortholog interacts with other transcription factors of known (AT1G03040) and unknown (AT5G03500, AT5G03495) function, a protein involved in phosphate starvation (SPX4), a protein of phytochrome response (PAR1), as well as proteins of unknown functions (AT3G27420, AT5G11980, AT4G24840). Based on the homology between UNE12 and rp11_olive_029403, rp11_olive_029403 likely is a good candidate for a transcription factor regulating reproductive tissues.

### The Reproductive Transcriptome According to ReprOlive

#### ReprOlive is a Complementary Source of Information for Olive Tree Transcriptome

The ReprOlive reproductive transcriptome includes a significantly higher number of final TTs than those provided by most studies (Alagna et al., 2009; Galla et al., 2009; Ozgenturk et al., 2010), although lower than the reported by Muñoz-Merida et al. (2013) resulting from the screening of numerous vegetative tissues and stages. The TT mean length is significantly higher than previous studies likely due to the use of Titanium+ technology. As a result, ReprOlive TTs are highly complementary to previous studies, maybe representing the only publicly available annotated database fully dedicated to olive tree transcriptome, including tools for different types of search and functional and structural annotations. Some other publicly available databases including olive transcriptome sequences like NCBI (e.g., SRX193576 accession), Oleaestdb^11^ (Alagna et al., 2009), and the European Nucleotide Archive^12^ (e.g., SRR592583 accession), either include raw sequences only, or a lower degree of operative resources. In conclusion, ReprOlive may help researches devoted to either plant-reproduction or other disciplines to retrieve relevant information on the olive transcriptome.

#### Reproductive Transcriptome Expresses Approximately Half of Olive Tree Genes, Mainly in Pistil

As expected, pollen and pistil have TTs in common since (1) the number of TTs in reproductive transcriptome is below the sum of pollen and pistil transcriptomes (**Table 2**); (2) pistil and reproductive transcriptomes contain 146 pathways while pollen contains only 143; and (3) there are TTs whose expression data indicate that both are expressed in pollen and pistil. Row “Number of TTs with annotation” of **Table 2** shows that 87.8% TTs were functionally annotated, although not all annotated TTs contain an ortholog. The number of unique orthologs (**Table 2**, “Unique IDs” rows) indicates that pistil transcriptome seems to be more complex than the pollen transcriptome. Considering the number of orthologs with *A. thaliana* in TAIR10 and RefSeq (15,503 and 17,612, respectively), it can be suggested that the reproductive transcriptome is a subset ranging from 55 to 62% of the complete transcriptome if it is assumed that the olive tree genome, like *A. thaliana* (Lamesch et al., 2012), contains ∼27,200 protein-coding genes.

#### The High Proportion of Full-Length ORFs Reveals a Reliable Transcriptome

Sequencing and assembling where highly successful since the number of chimeras is very low and the number of complete ORFs is 12,6% of the transcriptome, and 24,8% of the unique IDs, which is clearly above other transcriptomes built with the same strategy (Benzekri et al., 2014; Canales et al., 2014). Having a large collection of full-length protein sequences is crucial for accurate annotation, comparative analysis between transcriptomes, and also for obtaining accurate gene expression profiles related to growth, development, and environmental changes. In fact, an important collection of those full-length ORFs [such as the numerous forms of the Ole e 1 and Ole e 2 (profilins) allergens, Cu,Zn-superoxide dismutases, catalases, peroxidases, NADPH oxidase, enzymes of the glutathion-ascorbate cycle and thioredoxins] have been cloned by the authors based on the sequence in the database (results not shown), which confirms their reliability and utility.

#### Tentative Transcripts without Ortholog as a Source of Putative New Olive Specific Transcripts

Even though the Full-LengtherNext analysis shown in **Table 2** (column “Reproductive”) is quite strict in assigning an ortholog, 63,965 TTs were annotated and only 17,445 TTs remain unknown. The TransDecoder analysis contained in Full-LengtherNext, which can identify proteins in an orthology-independent way, revealed that 7,114 TTs of the 17,445 TTs with an unidentified ortholog could code for a protein, suggesting that 10,331 TTs could be discarded from the reproductive transcriptome. From the likely coding TTs, 628 of them code for a complete protein and 6,486 for an incomplete one. After the functional annotation using Sma3, 80 (12.7%) of likely complete TTs and 1,750 (27%) of likely incomplete TTs remain with no functional annotation at all, and other 134 (21.3%) and 1,612 (24.8%), respectively, are annotated as “uncharacterized” protein. These subsets of coding TTs should include some kind of olive-specific TTs, opening new research opportunities in olive tree for deciphering their function.

#### Reference Transcriptomes Seem to Gather the Olive Tree Heterozygosis

The size of transcriptomes is over-representing the putative number of olive tree genes, representing the maximal number of TTs expressed in reproductive tissues tested in this manuscript. This overestimation may come from the presence of alleles, paralogues, fragmented sequences, alternative splicing, and even a combination of them. Therefore, a subset of the transcriptome including the longest TTs with unique, different orthologous ID, and the longest, >500 bp, non-redundant unknown TT with coding or putative coding status is provided as a kind of “Reference transcriptome” (**Table 2**. It is useful for expression studies, such as expression analyses included in the “Expression” panel of the database. Sequences belonging to a reference transcriptome are easily identified by a “REF” tag (**Figures 2** and **3**) on their description. Since the numbers of unique RefSeq and TAIR10 IDs in complete (17,612 and 15,503, respectively) and reference (14,706 and 13,584, respectively) reproductive transcriptomes are quite close, it is suggested that, even if some genes could be lost, the Reference Transcriptome is representative of genes expressed in reproductive tissues. Moreover, two alleles for every locus seem to be included in the Reference Transcriptomes since the number of unique TAIR10/RefSeq IDs is ∼65% of the total TAIR10/RefSeq IDs (**Table 2**, rows below “Reference transcriptome”).

## Conclusion and Future Prospects

ReprOlive offers transcriptomic information related to olive tree reproductive tissues (with leaf and root as vegetative control) with unrestricted public access. It contains sufficient information on TTs that can be used for genomics, molecular studies, genetic maps, expression analyses, new allergen detection, and even future breeding purposes. It offers a comprehensive on-line system for information retrieval and management, and has help in the mining of reproductive transcriptome. The availability of a reference transcriptome with preliminary expression data and putative olive-specific genes give chances to new research areas. ReprOlive has also been published as a standard semantic conceptualization in RDF, enabling its integration with other RDF-based databases to provide distributed, updated annotation as well as data integration. Thus, ReprOlive joins the most novel approach to publish Open Data as previously done by relevant databases as UniProtKB^13^. In a near future, more olive sequences from public resources and our own research studies will be collected and archived, including future RNA-seq data sets, in order to provide the most complete information about the overall olive tree transcriptome. This may include other reproductive cells, tissues and organs of interest (such as isolated meiocytes, tapetum, endosperm, mesocarp, ovary, and embryo sac), as well as additional developmental stages and olive cultivars of interest. Therefore, ReprOlive (both in its present form and thorough future developments) may help researches devoted to either plant-reproduction or other disciplines to retrieve relevant information on olive transcriptome. Moreover, the TTs described here may be very helpful for complementing or corroborating the genome annotation in OLEA and IOGC genome projects. Full integration of olive databases is a goal to be pursued by all the consortia involved in these developments, and will be considered in future versions of ReprOlive.

## Conflict of Interest Statement

The authors declare that the research was conducted in the absence of any commercial or financial relationships that could be construed as a potential conflict of interest.

## Footnotes

1 http://www.oleagenome.org

2 http://olivegenome.karatekin.edu.tr

3 http://www.ncbi.nlm.nih.gov/bioproject/287107

4 http://www.scbi.uma.es/seqtrimnext

5 www.ncrna.org

6 http://transdecoder.github.io

7 http://www.scbi.uma.es/fulllengthernext

8 http://bioinfo.lifl.fr/mreps/

9 http://bioinfo.lifl.fr/mreps/

10 http://150.214.214.6/olivedb.owl

11 http://140.164.45.140/oleaestdb/index.php

12 http://www.ebi.ac.uk/ena/home

13 http://beta.sparql.uniprot.org/sparql/

## Supplementary Material

Figure S1 **Flow diagram of the strategy for pre-processing, assembling and annotation of the transcriptomes described in this manuscript, where yellow, double-lined boxes are the inputs, and the black boxes provide the output results**.

Figure S2 **The flow template based on AutoFlow that automates the complete process from pre processing to annotation.** Execution AutoFlow with the parameter –graphic with this flow template produces its semantic representation as in **Supplementary Figure S1**.

Figure S3 **GO enrichment using GOrilla of the 1655 TTs that are pollen-specific, sorted by their RPKM, that have an *Arabidopsis* orthologue in RefSeq.** The upper part contains the significant biological processes and the lower part reveals the cellular component where the processes occur.
