# Supplementary material for: ReprOlive: a database with linked data for the olive tree (Olea europaea L.) reproductive transcriptome
Source: Front Plant Sci. 2015 Aug 11;6:625. doi: 10.3389/fpls.2015.00625 (PMC4531244; doi:10.3389/fpls.2015.00625)
Supplement: Figure S1 — Flow diagram of the strategy for pre-processing, assembling and annotation of the transcriptomes described in this manuscript, where yellow, double-lined boxes are the inputs, and the black boxes provide the output results. [file Image_1.PDF]

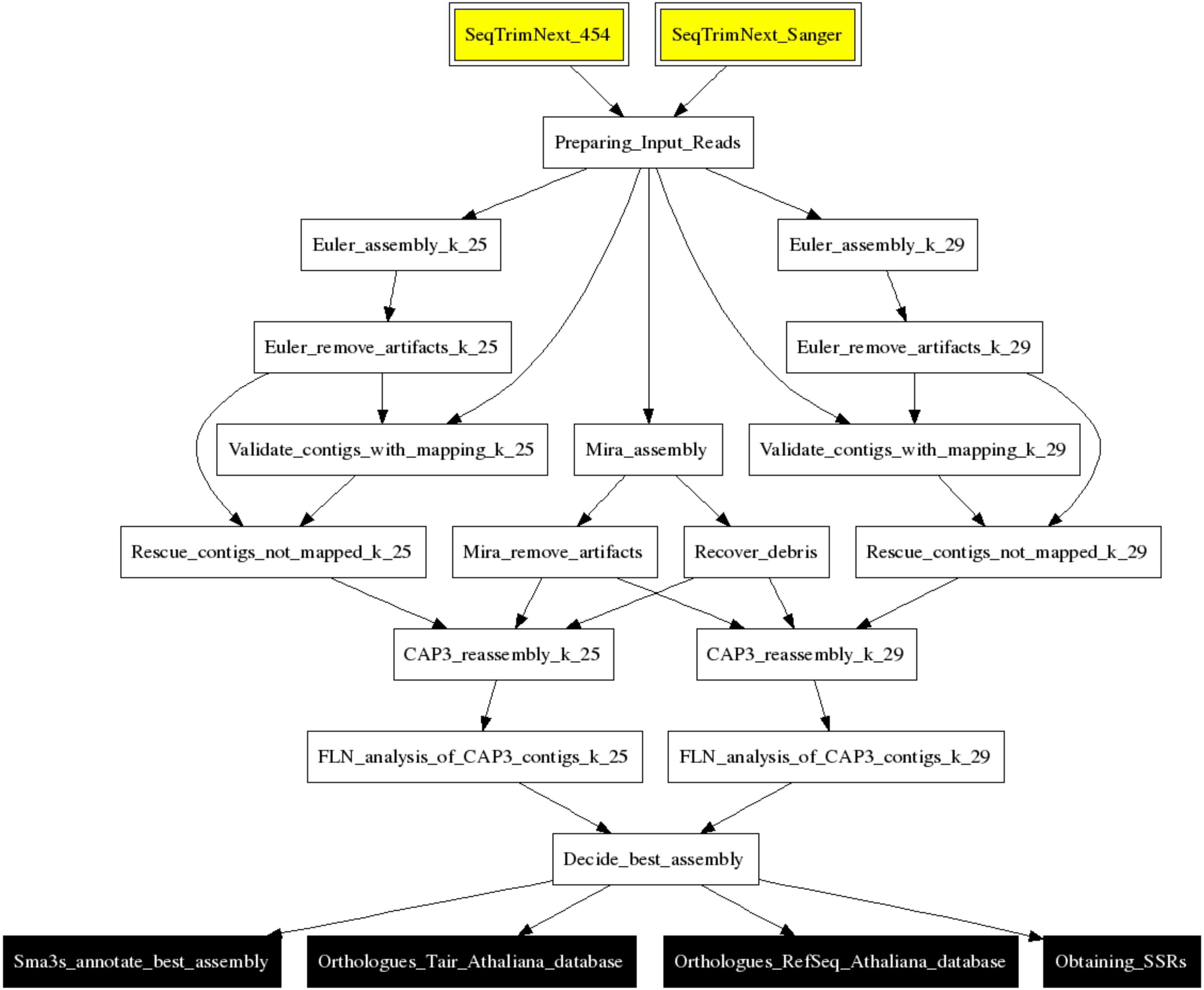

Supplementary File 1: Flow diagram of the strategy for pre-processing, assembling and annotation of the transcriptomes described in this manuscript where yellow, double-lined boxes are the inputs, and the black boxes are the output results
