# Supplementary material for: ReprOlive: a database with linked data for the olive tree (Olea europaea L.) reproductive transcriptome
Source: Front Plant Sci. 2015 Aug 11;6:625. doi: 10.3389/fpls.2015.00625 (PMC4531244; doi:10.3389/fpls.2015.00625)
Supplement: Figure S2 — The flow template based on AutoFlow that automates the complete process from pre processing to annotation. Execution AutoFlow with the parameter –graphic with this flow template produces its semantic representation as in Supplementary Figure S1. [file Image_2.PDF]

```
########################################################################################################################
# Declaring variables
########################################################################################################################

# static variables
$original_454_reads=../../454_reads.fastq
$original_sanger_reads=../../Sanger_reads.fastq
$assembly_name=reproductive
$transcript_name=rp11_olive_
$db_orthologues_path=../../
$db_orthologues=[Tair_Athaliana_database;RefSeq_Athaliana_database]

# dynamic variable where the best assembly path will be stored
@best_assembly=NULL

########################################################################################################################
# Pre-processing 454/Roche reads using SeqTrimNext
########################################################################################################################
SeqTrimNext_454){
    module load seqtrimnext/last
    ?
    seqtrimnext -t transcriptomics_454_plants.txt -Q $original_454_reads -w [lcpu] -s 10.243 > stn_454_reads.txt
    cat output_files/RL*/sequences*.fastq > input_mira_in.454.fastq
}

########################################################################################################################
# Pre-processing Sanger reads using SeqTrimNext
########################################################################################################################
SeqTrimNext_Sanger){
    module load seqtrimnext/last
    ?
    seqtrimnext -t sanger.txt -Q $original_sanger_reads -w [lcpu] -s 10.243 > stn_sanger_reads.txt
    mv output_files/sequences_.fastq input_mira_in.sanger.fastq
}

########################################################################################################################
# Preparing reads for assembling
########################################################################################################################
Preparing_Input_Reads){
    module load seqtrimnext/last
    ln -s SeqTrimNext_454)/input_mira_in.454.fastq
    ln -s SeqTrimNext_Sanger)/input_mira_in.sanger.fastq
    ?
    fastq2fasta.rb SeqTrimNext_454)/input_mira_in.454.fastq input_euler_454
    fastq2fasta.rb SeqTrimNext_Sanger)/input_mira_in.sanger.fastq input_euler_sanger
    cat input_euler_454.fasta input_euler_sanger.fasta > input_euler.fasta
}

########################################################################################################################
#Assembling with MIRA3 (OLC assembler)
########################################################################################################################

Mira_assembly){
    module load mira/3.2.0
    ln -s Preparing_Input_Reads)/input_mira_in.454.fastq
    ln -s Preparing_Input_Reads)/input_mira_in.sanger.fastq
    ln -s Preparing_Input_Reads)/input_euler.fasta
    mkdir -p $SCRATCH/$assembly_name
    ?
    mira -fastq -project=input_mira --job=denovo,est,normal,454,sanger -CL:ascdc  SANGER_SETTINGS -CO:fnicpst=yes
454_SETTINGS  -CO:fnicpst=yes -notraceinfo  COMMON_SETTINGS -GE:not=4 -DI:lrt=$SCRATCH/$assembly
    rm -rf $SCRATCH/$assembly_name

}

 # Remove artifacts from MIRA3
Mira_remove_artifacts){
    source ~soft_cvi_114/initializes/init_fln
    gem list full_lengther_next
    sort_fasta_list.rb Mira_assembly)/input_mira_assembly/input_mira_d_results/input_mira_out.unpadded.fasta
    lista_to_fasta.rb Mira_assembly)/input_mira_assembly/input_mira_d_results/input_mira_out.unpadded.fasta list >
mira_contigs_dispersed.fasta
    ?
```

```
    full_lengther_next -f mira_contigs_dispersed.fasta -g plants -c 500 -z -w [lcpu]
}

 # Recover sequences from debris of MIRA3
Recover_debris){
    source ~soft_cvi_114/initializes/init_fln
    lista_to_fasta.rb Mira_assembly)/input_euler.fasta
Mira_assembly)/input_mira_assembly/input_mira_d_info/input_mira_info_debrislist.txt > mira_debris.fasta
    sort_fasta_list.rb mira_debris.fasta
    lista_to_fasta.rb mira_debris.fasta list > mira_debris_dispersed.fasta
    ?
    full_lengther_next -f mira_debris_dispersed.fasta -g plants -c 500 -z -w [lcpu] -q d
    table_header.rb -t fln_results/pt_seqs n > coding_debris_mira.list
    table_header.rb -t fln_results/new_coding.txt >> coding_debris_mira.list
    lista_to_fasta.rb mira_debris_dispersed.fasta coding_debris_mira.list > coding_debris_mira.fasta
}

###########################################################################################################
# Assembling with EULER (de Bruijn assembling using 25 & 29 k-mers)
###########################################################################################################
Euler_assembly_k_[25;29]){
    module load euler/120408
    ln -s Preparing_Input_Reads)/input_euler.fasta
    ?
    Assemble.pl input_euler.fasta (*)
}

# Remove artifacts from EULER
Euler_remove_artifacts_k_[25;29]){
    source ~soft_cvi_114/initializes/init_fln
    sort_fasta_list.rb !Euler_assembly_k_*!/input_euler.fasta.contig
    lista_to_fasta.rb !Euler_assembly_k_*!/input_euler.fasta.contig list > dispersed_input_euler.fasta
    ?
    full_lengther_next -f dispersed_input_euler.fasta -g plants -c 500 -z -w [lcpu]
}


Validate_contigs_with_mapping_k_[25;29]){
    module load bowtie/v2_2.0.0-beta7
    bowtie2-build -f !Euler_remove_artifacts_k_*!/fln_results/unigenes.fasta ref
    ?
    bowtie2 ref -f -U Preparing_Input_Reads)/input_euler.fasta -p [cpu] --very-fast -S mapeo.sam
}


Rescue_contigs_not_mapped_k_[25;29]){
    module load samtools/0.1.16
    ?
    mapping_tool.rb -i !Validate_contigs_with_mapping_k_*!/mapeo.sam -f -o euler_mapped_contigs.list
    mapping_tool.rb -i !Validate_contigs_with_mapping_k_*!/mapeo.sam -f -r -o euler_unmapped_contigs.list
    lista_to_fasta.rb !Euler_remove_artifacts_k_*!/fln_results/unigenes.fasta euler_mapped_contigs.list >
euler_mapped_contigs.fasta
    lista_to_fasta.rb !Euler_remove_artifacts_k_*!/fln_results/unigenes.fasta euler_unmapped_contigs.list > temp.fasta
    table_header.rb -t !Euler_remove_artifacts_k_*!/fln_results/pt_seqs > annot_coding_euler_unmapped.list
    table_header.rb -t !Euler_remove_artifacts_k_*!/fln_results/new_coding.txt >> annot_coding_euler_unmapped.list
    lista_to_fasta.rb temp.fasta annot_coding_euler_unmapped.list > euler_coding_unmapped_contigs.fasta
}

############################################################################################################
## CAP3 (final reconciliation of MIRA3 contigs with each Euler-25 and Euler-29 contigs)
############################################################################################################

CAP3_reassembly_k_[25;29]){
    module load cap3/101507
    ?
    cat Recover_debris)/coding_debris_mira.fasta Mira_remove_artifacts)/fln_results/unigenes.fasta
!Rescue_contigs_not_mapped_k_*!/euler_mapped_contigs.fasta
!Rescue_contigs_not_mapped_k_*!/euler_coding_unmapped_contigs.fasta  > reassembly.fasta
    cap3 reassembly.fasta -p 95 -o 40
}

FLN_analysis_of_CAP3_contigs_k_[25;29]){
   module load ruby
   source ~soft_cvi_114/initializes/init_fln
```

```
   cat !CAP3_reassembly_k_*!/reassembly.fasta.cap.contigs !CAP3_reassembly_k_*!/reassembly.fasta.cap.singlets >
unigenes.fasta
   sort_fasta_list.rb unigenes.fasta
   lista_to_fasta.rb unigenes.fasta list > dispersed_unigenes.fasta
   fasta_standard_renamer.rb dispersed_unigenes.fasta $transcript_name 6
   ?
   full_lengther_next -f dispersed_unigenes.fasta_new -g plants -c 300 -z -r -w [lcpu]
}

##############################################################################################################################
## Deciding the best assembly
##############################################################################################################################

Decide_best_assembly){
    module load ruby
    assembly=`fln_assembly_evaluator !FLN_analysis_of_CAP3_contigs_k_! `
    ?
    echo $assembly
    env_manager "best_assembly=$assembly;"
}

##############################################################################################################################
## Annotating the best assembly with sma3s
##############################################################################################################################

Sma3s_annotate_best_assembly){
    . ~soft_cvi_114/initializes/init_distributed_sma3s
    ?
    distributed_sma3s -c 'sma3s_v2.pl -a 123 -p F -v 2' -i $best_assembly/fln_results/unigenes.fasta -o olive.annot -d
/mnt/home/soft/blast_db/current/fmt/sma3/uniprot_plants.dat -g 250 -w [lcpu]
}

###############################################################################################################################
# Obtaining ORTHOLOGUES in others Arabidopsis thaliana databases
###############################################################################################################################
Orthologues_$db_orthologues){
    source ~soft_cvi_114/initializes/init_fln
    echo Decide_best_assembly)
    mkdir db
    makeblastdb -in $db_orthologues_path/(*) -dbtype prot -parse_seqids -out db/(*)
    ?
    full_lengther_next -f $best_assembly/fln_results/unigenes.fasta -a '' -u db/(*) -c 500 -z -w [lcpu] -q d -g plants
}

###############################################################################################################################
# Obtaining SSRS (microsatellites)
###############################################################################################################################
Obtaining_SSRs){
    module load ruby
    module load mreps/2.5
    echo Decide_best_assembly)
    ln -s $best_assembly/fln_results/unigenes.fasta
    ?
    replace_n.rb unigenes.fasta
    rm_codigo_degenerado.rb replaced_n_unigenes.fasta
    mreps -minsize 12 -minperiod 2 -exp 3.0 -fasta new_replaced_n_unigenes.fasta > $assembly_name_ssr.txt
}

###############################################################################################################################
```
