# Supplementary figures and images for: ReprOlive: a database with linked data for the olive tree (Olea europaea L.) reproductive transcriptome
Source: Front Plant Sci. 2015 Aug 11;6:625. doi: 10.3389/fpls.2015.00625 (PMC4531244; doi:10.3389/fpls.2015.00625)

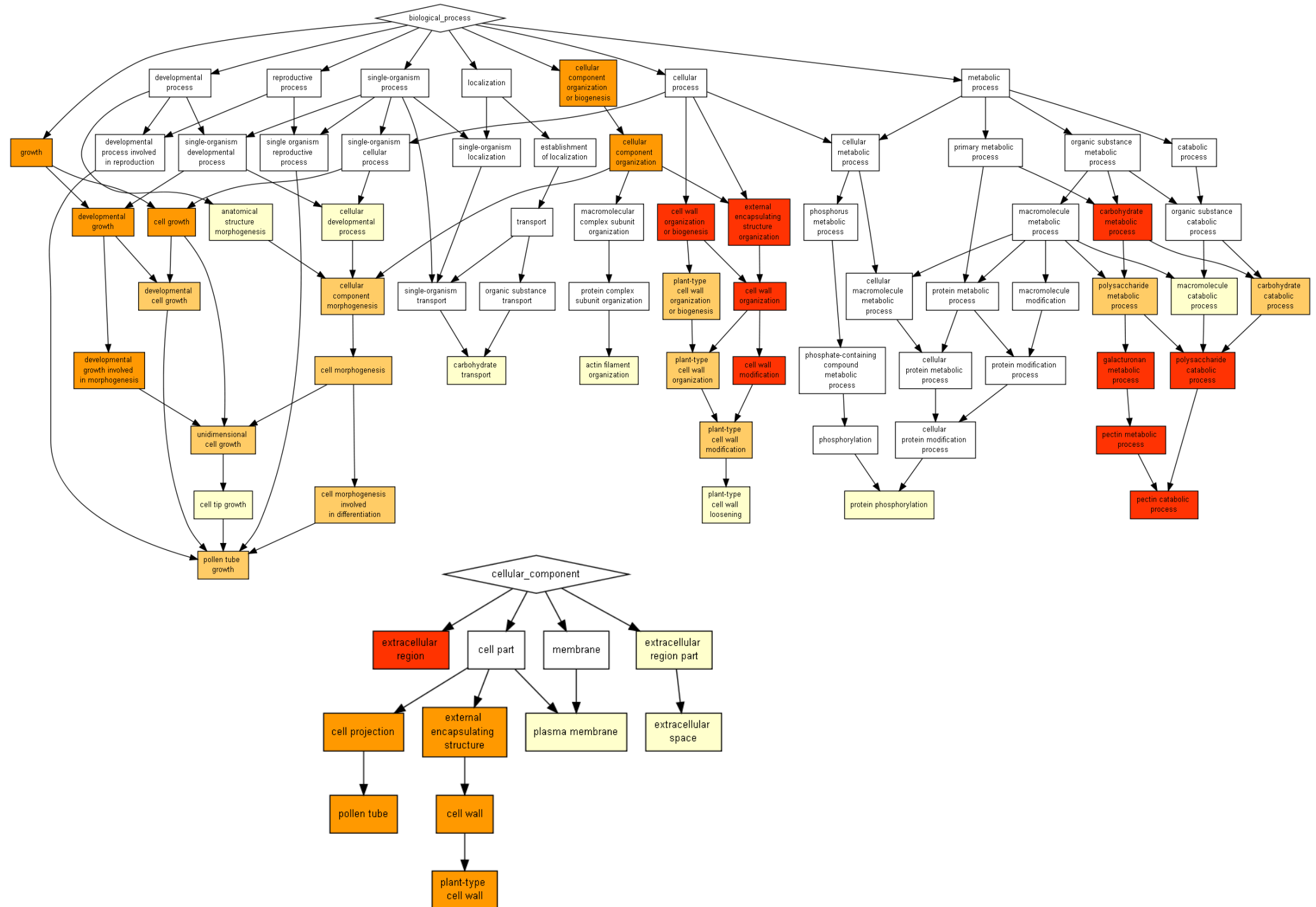

Supplement: Figure S3 — GO enrichment using GOrilla of the 1655 TTs that are pollen-specific, sorted by their RPKM, that have an Arabidopsis orthologue in RefSeq. The upper part contains the significant biological processes and the lower part reveals the cellular component where the processes occur. [file Image_3.PDF]
